# Theta burst stimulation over left cerebellum does not modulate auditory feedback control of vocal production

**DOI:** 10.3389/fnins.2022.1051629

**Published:** 2022-12-22

**Authors:** Dongxu Liu, Mingyun Chen, Qing Lin, Tingni Li, Xi Chen, Guangyan Dai, Xiuqin Wu, Jingting Li, Hanjun Liu, Peng Liu

**Affiliations:** ^1^Department of Rehabilitation Medicine, The First Affiliated Hospital, Sun Yat-sen University, Guangzhou, China; ^2^Department of Rehabilitation Medicine, Zhongshan Hospital Affiliated to Xiamen University, Xiamen, China; ^3^Guangdong Provincial Key Laboratory of Brain Function and Disease, Zhongshan School of Medicine, Sun Yat-sen University, Guangzhou, China

**Keywords:** left cerebellum, speech motor control, auditory feedback, theta burst stimulation, event-related potential

## Abstract

**Background:**

Accumulating evidence has shown significant contributions of the right cerebellum to auditory-motor integration for vocal production. Whether the left cerebellum is likewise involved in vocal motor control, however, remains unclear.

**Methods:**

By applying neuronavigated continuous and intermittent theta burst stimulation (cTBS/iTBS) over the left cerebellar lobule VII (Crus I), the present event-related potential (ERP) study investigated whether the left cerebellum exerts causal effects in modulating auditory feedback control of vocal pitch production. After receiving cTBS, iTBS, or sham stimulation over the left cerebellum, a group of fifteen young adults produced sustained vowels while hearing their voice unexpectedly shifted in pitch upwards or downwards by 200 cents. The effects of cerebellar stimulation were assessed by measuring the vocal and ERP (N1/P2) responses to pitch perturbations across the conditions.

**Results:**

When compared to sham stimulation, cTBS or iTBS over the left cerebellar lobule VII (Crus I) led to no systematic changes in vocal compensations for pitch perturbations in auditory feedback. Also, the cortical N1/P2 responses did not vary significantly across the three stimulation sessions.

**Conclusion:**

These findings present the first neurobehavioral evidence suggesting that the left cerebellum is not causally associated with auditory feedback control of vocal production. Together with previously reported causal effects of the right cerebellum in modulating vocal pitch regulation, the present study lends support to the hypothesis that there is a functional lateralization of the cerebellum in vocal motor control though auditory feedback.

## Introduction

The integration of auditory feedback and motor systems is one fundamental aspect of speech production, supporting the generation of the desired speech accurately through the online detection and correction of mismatches between intended and actual vocal output (Hickok et al., 2011). By perturbing fundamental frequency (*f*_*o*_) or formant frequency (*F*_1_) in auditory feedback during vocal/speech production, a growing body of literature has shown that auditory-vocal integration receives significant contributions from the cerebellum, one substructure that has been long considered to be essential in coordinating limb movement and motor functions (Manto et al., 2012). For example, increased cerebellar activity has been identified during the generation of compensatory speech responses to *F*_1_ perturbations in healthy individuals (Tourville et al., 2008). Clinical studies on patients with spinocerebellar ataxia (SCA) have shown abnormally reduced adaptive responses to predictable speech *F*_1_ perturbations but enhanced corrective responses to unexpected vocal pitch perturbations (Parrell et al., 2017; Houde et al., 2019; Li et al., 2019). More recently, two non-invasive brain stimulation studies provide causal evidence that supports cerebellar contributions to vocal motor control through auditory feedback, as reflected by increased vocal compensations for pitch perturbations in healthy individuals following cerebellar anodal transcranial direct current stimulation (tDCS) (Peng et al., 2021) and decreased vocal compensations in patients with SCA following cerebellar continuous theta burst stimulation (cTBS) (Lin et al., 2022). These findings have implicated an essential role for the cerebellum in auditory-motor integration for speech/vocal production.

Nevertheless, one important question that remains open is whether the cerebellum is unilaterally or bilaterally involved in auditory-vocal integration. Most of previous studies have shown functional associations between the right cerebellum and speech/language production. Neuroimaging studies have revealed activation of the right cerebellum during verbal generation tasks (Riecker et al., 2000; Stoodley, 2012), verbal working memory (Riva and Giorgi, 2000), voiced speech (Schulz et al., 2005), and compensatory adjustment of speech *F*_1_ (Tourville et al., 2008). More importantly, a series of tDCS and cTBS studies have shown a causal relationship between the right cerebellum and language performance or speech motor control. For example, applying anodal tDCS and cTBS over the right cerebellum respectively resulted in increased (Peng et al., 2021) and decreased (Lin et al., 2022) vocal compensations for pitch perturbations. Increased speech compensations for *F*_1_ perturbations were found when anodal tDCS was applied over the right cerebellum (Lametti et al., 2018). In addition, cTBS over the right cerebellum impaired verbal working memory and reduced accuracy in lexical tasks, whereas cTBS over the contralateral region did not show the same effect (Argyropoulos, 2011; Tomlinson et al., 2014). Clinically, impaired speech articulation and planning are generally associated with damage to the right cerebellum (Silveri et al., 1998; Ackermann et al., 2007), and anodal and/or cathodal tDCS over this region coupled with language treatment can improve verbal generation or picture naming in chronic post-stroke patients with aphasia (Marangolo et al., 2018; Sebastian et al., 2020).

In contrast, there is limited evidence suggesting that the left cerebellum may be also involved in speech/language production. For example, activation of the bilateral cerebellar hemisphere was found during sequence syllable production (Bohland and Guenther, 2006), vocalization of a single pitch (Perry et al., 1999), and articulatory control tasks (Chen and Desmond, 2005). Also, previous meta-analysis studies revealed significant contributions of bilateral cerebellum to word reading (Turkeltaub et al., 2002; Indefrey and Levelt, 2004). In addition, language deficits including word dysfluency and sentence formulation were reported in patients with the left primary cerebellar lesions (Cook et al., 2004; Murdoch and Whelan, 2007), and motor speech disorders such as dysarthria resulted more frequently from damage to the left than right cerebellum (Amarenco et al., 1991). In the context of speech motor control, activation of bilateral cerebellum was identified when somatosensory feedback was perturbed during speech production (Golfinopoulos et al., 2011). These findings suggest that, in addition to the right cerebellum, the left cerebellum may also be a significant contributor to sensorimotor control of speech production. Unfortunately, there is by far no direct causal evidence for this brain-behavior relationship.

To this end, the present event-related potential (ERP) study investigated the neurobehavioral correlates of auditory-motor integration for vocal pitch regulation by modulating activity of the left cerebellum with cTBS and intermittent TBS (iTBS). TBS is a specific form of transcranial magnetic stimulation (TMS), where cTBS generally suppresses neuronal excitability while iTBS induces the opposite effects (Huang et al., 2011). After receiving cTBS, iTBS, or sham stimulation over the left cerebellum, the participants vocalized the vowel sounds while hearing their voice pitch-shifted using the frequency-altered feedback (FAF) paradigm (Burnett et al., 1998). The neurobehavioral effects were assessed by measuring the vocal and ERP responses (N1 and P2) to pitch perturbations; these parameters have been successfully used to probe the causal relationship between certain brain region and vocal motor control (Liu et al., 2020; Li et al., 2022). Our results showed no systematic changes of vocal and N1/P2 responses to pitch perturbations across the three stimulation sessions, suggesting a lack of causal evidence that supports the involvement of the left cerebellum in auditory-vocal integration.

## Materials and methods

### Subjects

Fifteen right-handed, native-Mandarin speakers (seven female and eight male; age: 21.06 ± 1.09 years), who were college students from Sun Yat-sen University of China, participated in the present study. They had no history of pregnancy, speech or hearing disorders, implanted medical device, intake of psychiatric or neurological medication, or formal musical training. One female participant was excluded from the final analyses due to the poor quality of her vocal data. Therefore, the present study included the data from 14 participants (six female and eight male; age: 21.43 ± 1.09 years). Written informed consent was obtained from each participant, and the research protocol was approved by the Institutional Review Board of The First Affiliated Hospital of Sun Yat-sen University.

### Neuronavigated transcranial magnetic stimulation

Prior to the TMS experiment, all participants underwent a high-resolution structural MRI in a 3T scanner (Siemens, Erlangen, Germany) to determine the target site. During the scanning, a T1-weighted magnetization-prepared rapid gradient-echo (MPRAGE) sequence was used with the following parameters: repetition time = 2300 ms, echo time = 2.19 ms, slice thickness = 1 mm, field of view = 256 × 256 mm^2^, flip angle = 9.

**FIGURE 1:**
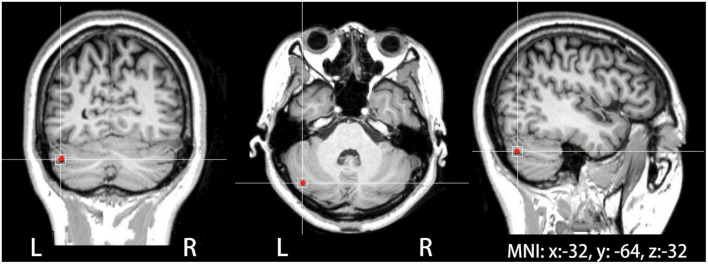
The site of theta burst stimulation (TBS) stimulation located in the left cerebellar lobule VII (Crus I), which was identified on individual MRIs in coronal, axial, and sagittal views of the brain with the help of a neuronavigation system.

Transcranial magnetic stimulation was administered with a 7 cm-outer-diameter figure-of-eight coil connected to a CCY-I TMS instrument (YIRUIDE Co., Wuhan, China). Single-pulse TMS was applied over the right primary motor cortex to determine active motor threshold (AMT), defined as the lowest intensity inducing motor-evoked potentials (MEPs) of at least ≥200 μV in 5 out of 10 trials during 10% of maximum contraction of the left first dorsal interosseous muscle (Rossi et al., 2009). In the present study, TMS was delivered to the target site at 80% of AMT (Lin et al., 2022). Neuronavigated TMS was performed to localize the target site and monitor the coil position using a neuronavigation software (Visor2, ANT Neuro, Netherlands) with a Polaris Spectra motion tracking system (NDI, Canada). The target site was localized using the mean Montreal Neurological Institute (MNI) coordinates of the left cerebellar lobule VII (Crus I) (*x*: −32, *y*: −64, *z*: −32) (see Figure 1), contralateral to the right cerebellar lobule VII (Crus I) that was found to be involved in auditory feedback control of vocal production (Lin et al., 2022). These coordinates were slightly modified based on individual brain anatomical landmarks if necessary.

The present study included three stimulation sessions: cTBS over the left cerebellum, iTBS over the left cerebellum, and sham cTBS/iTBS over the left cerebellum. A standard TBS protocol was applied over the target site for each participant, where cTBS consisted of three-pulses bursts at 50 Hz repeated every 200 ms for 40 s while iTBS consisted of three-pulses bursts at 50 Hz repeated every 200 ms for a total of 600 pules (Huang et al., 2011). The plane of the coil was tangent to the scalp during active stimulation, while the place of the coil was perpendicular to the tangent plane of the target site with the edge of the coil touching the scalp during sham stimulation. Half of the participants received sham cTBS over the left cerebellum and the other half received sham iTBS over the left cerebellum. The three stimulation sessions were conducted in a counterbalanced manner across all participants, occurring on separate days at least 7 days apart.

### Frequency-altered feedback experiment

**FIGURE 2:**
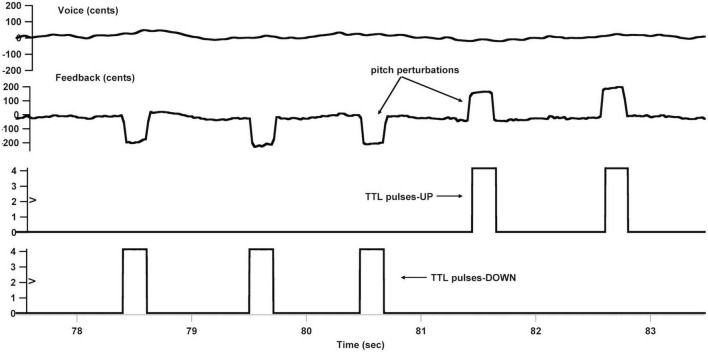
Overview of the frequency-altered feedback (FAF) paradigm. From top to bottom: voice *f*_o_ contour in cents, auditory feedback containing pitch perturbations in cents, and transistor-transistor logic (TTL) control pulses that signaled the onset of upward and downward pitch perturbations.

An FAF-based vocal production experiment was conducted immediately following active or sham left cerebellar TBS for all participants to maximize the after-effects. They were instructed to vocalize the /u/sound at their habitual pitch and loudness levels and maintained it steady for approximately 5-6 s, during which their voice was pseudo-randomly pitch-shifted five times by +200 cents or −200 cents (100 cents equal to one semitone) for 200 ms (see Figure 2). The first perturbation was presented with a random delay of 1200–1500 ms relative to the vocal onset, and the succeeding perturbations occurred with an inter-stimulus interval of 700–1,000 ms. The manipulation of the timing and direction of the pitch perturbations was to reduce the potential effects of implicit expectation on the neurobehavioral responses (Korzyukov et al., 2012). As well, previous studies showed directional effects of pitch perturbations on the vocal and/or ERP responses (Chen et al., 2007; Liu et al., 2011). Prior to initiating the next vocalization, the participants were required to take a break of 2–3 s to avoid the vocal fatigue. Each participant produced 40 consecutive vocalizations, leading to 200 trials that included 100 trials for +200 cents perturbations and 100 trials for −200 cents perturbations.

### Vocal and EEG data acquisition

The vocal production experiment was conducted in a sound-attenuated booth. The voice signals were picked up by a dynamic microphone (DM2200, Takstar Inc., Guangzhou, China) and amplified by a MOTU Ultralite Mk3 Firewire audio interface (Cambridge, MA). Then, they were sent to an Eventide Eclipse Harmonizer and pitch-shifted using a custom-developed MIDI software program (Max/MSP v.5.0 by Cycling 74, Walnut, CA, USA). This program also generated the transistor-transistor logic (TTL) control pulses to mark the onset of each perturbation. Finally, the pitch-shifted voice signals were amplified by an ICON NeoAmp headphone amplifier (Middleton, WI) and fed back to participants through insert earphones (ER-1, Etymotic Research Inc., Elk Grove Village, IL). The original and pitch-shifted voice signals as well as the TTL pulses were digitized by a PowerLab A/D converter (ML880, AD Instruments) and recorded at 10 kHz using LabChart software (v.7.0, AD Instruments, New South Wales, Australia).

The electroencephalography (EEG) signals were also picked up from the participant’s scalp using a 64-electrode Geodesic Sensor Net (Electrical Geodesics Inc., Eugene, OR, USA). After amplification by a Net Amps 300 amplifier (Electrical Geodesics Inc.), they were recorded at 1 kHz using NetStation software (v.4.5, Electrical Geodesics Inc.). The impedance levels of individual sensors were adjusted to be kept below 50 kΩ, since this amplifier allows the EEG data to be collected with high scalp-electrode impedances (40–60 kΩ) (Ferree et al., 2001). During the online recording, the EEG signals were referenced to the vertex (Cz) across all channels (Ferree et al., 2001). The TTL control pulses were sent to the EEG recording system *via* a DIN synch cable for synchronization of the voice and EEG signals.

### Vocal and EEG data analysis

As previously described (Li et al., 2019; Lin et al., 2022), a custom-developed IGOR PRO software program (v.6.0 by Wavemetrics Inc., Portland, OR, USA) was used to analyze the vocal responses to pitch perturbations. Briefly, the voice *f*_*o*_ contours in Hz were extracted from the acoustic signals and converted to cents according to the following formula: cents = 10 × (12 × log2[*f*_*o*_/reference]) [reference = 195.997 Hz (G3 note)]. The voice *f*_*o*_ contours in cents were then segmented into epochs ranging from −100 to +700 ms relative to the perturbation onset and visually inspected to remove those trials that contained artifacts arising from signal processing errors or unexpected voice stops. Finally, all artifact-free trials were averaged and baseline-corrected (−100 to 0 ms) to generate an overall vocal response. The peak magnitude of a vocal response was defined as the maximum or minimum value of the voice *f*_*o*_ contour in cents after the perturbation onset, and the peak time was measured as the time in milliseconds when the voice *f*_*o*_ contour reached its peak value.

The EEG signals were analyzed offline using NetStation software. They were band-pass filtered at 1–20 Hz, segmented into epochs ranging from −200 to +500 ms relative to the perturbation onset, and inspected using an artifact detection procedure. The trials were marked bad and excluded from further analysis if they exceeded ±55 μv of the moving average over an 80 ms window or contained more than 10 bad channels. An additional visual inspection was performed to ensure that all bad trials were appropriately rejected. Finally, all artifact-free trials were re-referenced to the average of the electrodes on each mastoid, averaged, and baseline-corrected (−200 to 0 ms) to generate an overall ERP response. Three regions of interest (ROI) that included 24 electrodes were defined for statistical analysis (Liu et al., 2020; Lin et al., 2022): frontal area, including AF3, AFz, AF4, F5, F3, F1, Fz, F2, F4, F6; fronto-central area, including FC5, FC3, FC1, FCz, FC2, FC4, FC6; central area, including C5, C3, C1, Cz, C2, C4, C6. The amplitudes and latencies of the N1 and P2 components were defined as the negative and positive peak values and times in the time windows of 80–180 and 160–280 ms and extracted from the averaged ERPs for each ROI, respectively.

### Statistical analysis

The values of vocal and ERP (N1 and P2) responses were analyzed using repeated-measures analysis of variances (RM-ANOVAs) in SPSS (v.20.0). The peak magnitudes and times of vocal responses were subjected to two-way RM-ANOVAs, including factors of perturbation direction (+200 and −200 cents) and stimulation session (cTBS, iTBS, and sham). The amplitudes and latencies of the N1 and P2 responses were subjected to three-way RM-ANOVAs, including factors of stimulation session, perturbation direction and electrode site (frontal, frontal-central and central). Bonferroni adjustment was used in the post-hoc analyses for multiple comparison corrections. Greenhouse-Geisser corrected *p*-values were reported when the assumption of Mauchly’s test was violated. Differences across the conditions were considered significant when *p* < 0.05.

## Results

### Behavioral findings

**FIGURE 3:**
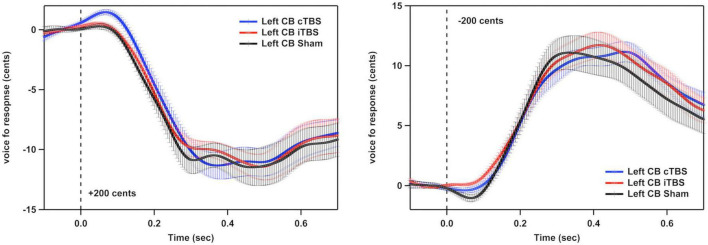
Grand-averaged voice *f*_o_ contours across all participants in responses to pitch perturbations of ±200 cents following continuous theta burst stimulation (cTBS) (blue), intermittent theta burst stimulation (iTBS) (red), and sham (black) stimulation over the left cerebellar lobule VII (Crus I). Time 0 represent the onset of pitch perturbations. CB, cerebellum.

**FIGURE 4:**
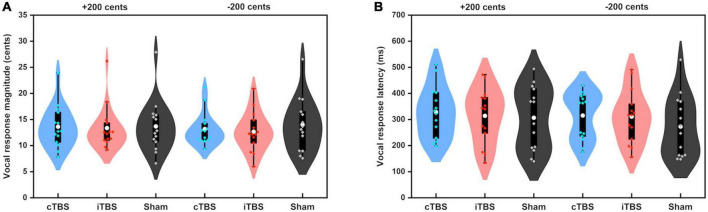
Violin plots of the peak magnitudes **(A)** and times **(B)** of vocal responses to ±200 cents produced by all participants across the conditions. The shape of the violin shows the kernel density estimate of the data. The white dots and box plots represent the medians and ranges from first to third quartiles of the data sets. The blue, red, and black dots represent the individual vocal responses to pitch perturbations following cTBS, iTBS, and sham stimulation over the left cerebellar lobule VII (Crus I). CB, cerebellum.

Figure 3 shows the grand-averaged voice *f*_*o*_ contours across all participants in responses to pitch perturbations of ±200 cents following cTBS, iTBS and sham stimulation over the left cerebellar lobule VII (Crus I). A two-way RM-ANOVA conducted on the peak magnitudes of vocal responses revealed no significant main effects of stimulation session [F(2, 26) = 0.510, *p* = 0.606] and perturbation direction [F(1, 13) = 0.274, *p* = 0.609] as well as their interaction [F(2, 26) = 0.544, *p* = 0.587] (see Figure 4A). Similarly, the peak times of vocal responses did not vary significantly as a function of stimulation session [F(2, 26) = 1.104, *p* = 0.347] and perturbation direction [F(1, 13) = 0.306, *p* = 0.589] (see Figure 4B). Their interaction was not significant either [F(2, 26) = 0.294, *p* = 0.747].

### ERP findings

**FIGURE 5:**
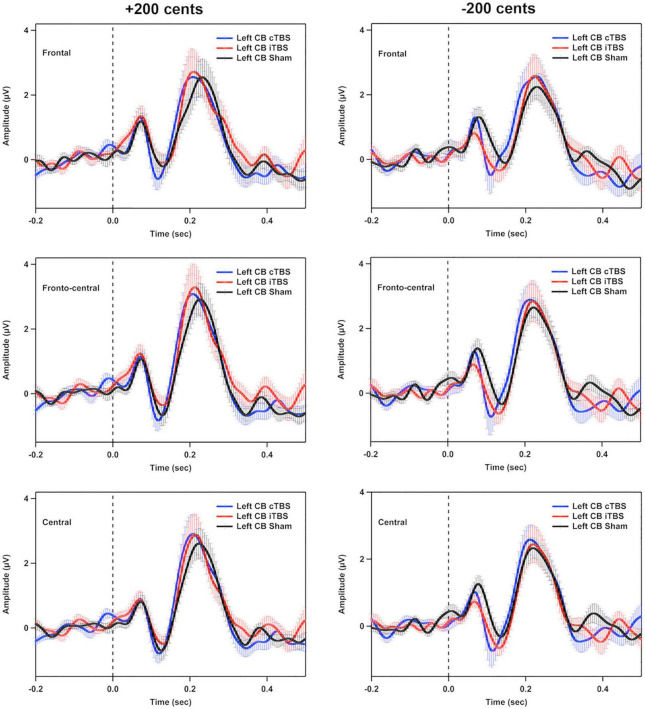
Grand-averaged event-related potential (ERP) waveforms across all participants in responses to pitch perturbations of ±200 cents in the frontal, fronto-central, and central regions following cTBS, iTBS, and sham stimulation over the left cerebellar lobule VII (Crus I). Time 0 represent the onset of pitch perturbations. CB, cerebellum.

**FIGURE 6:**
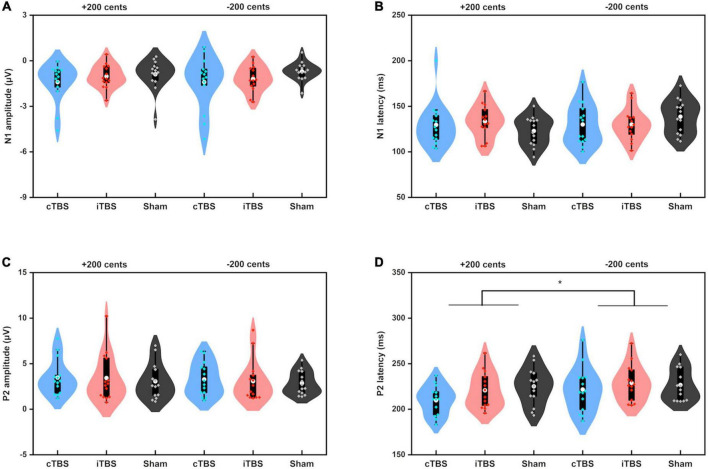
Violin plots of the amplitudes and latencies of the N1 **(A,B)** and P2 **(C,D)** responses to pitch perturbations of ±200 cents produced by all participants across the conditions. The white dots and box plots represent the medians and ranges from first to third quartiles of the data sets. The blue, red, and black dots represent the individual vocal responses to pitch perturbations following cTBS, iTBS, and sham stimulation over the left cerebellar lobule VII (Crus I). The asterisk indicates a significant difference in the P2 latency between +200 and −200 cents perturbations. CB, cerebellum.

Figure 5 shows the grand-averaged ERP waveforms across all participants in response to pitch perturbations of ±200 cents as a function of electrode site following cTBS, iTBS, and sham stimulation over the left cerebellar lobule VII (Crus I). A three-way RM-ANOVA conducted on the N1 amplitudes revealed no significant main effects of stimulation session [F(2, 26) = 1.697, *p* = 0.212], perturbation direction [F(1, 13) = 0.009, *p* = 0.928] and electrode site [F(2, 26) = 3.830, *p* = 0.056] (see Figure 6A). None of the interactions among any of three factors were found to be significant (*p* > 0.1). For the N1 latencies, the main effects of stimulation session [F(2, 26) = 0.061, *p* = 0.941], perturbation direction [F(1, 13) = 2.110, *p* = 0.170], and electrode position [F(2, 26) = 0.331, *p* = 0.626] as well as their interactions (*p* > 0.07) did not reach significance (see Figure 6B).

A three-way RM-ANOVA conducted on the P2 amplitudes revealed no significant main effects of stimulation session [F(2, 26) = 0.240, *p* = 0.788] and perturbation direction [F(1, 13) = 2.540, *p* = 0.135] (see Figure 6C). A significant main effect of electrode site [F(2, 26) = 6.201, *p* = 0.020) was found, indicating larger P2 amplitudes at the frontal-central electrodes than at the frontal (*p* = 0.032) and central electrodes (*p* < 0.001). The interactions among the three factors were not significant (*p* > 0.1). Regarding the P2 latencies, there was a significant main effect of perturbation direction [F(1, 13) = 12.398, *p* = 0.004], showing faster P2 responses to upward pitch perturbations than to downward pitch perturbations. However, the main effects of stimulation session [F(2, 26) = 2.119, *p* = 0.162] and electrode site [F(2, 26) = 3.790, *p* = 0.059] did not reach significance (see Figure 6D). In addition, there were no significant interactions among the three factors (*p* > 0.3).

## Discussion

By applying neuronavigated cTBS or iTBS over the left cerebellar lobule VII (Crus I), the present study investigated the functional association between the left cerebellum and auditory-vocal integration in a causal manner. When compared to sham stimulation, cTBS or iTBS over the left cerebellar lobule VII (Crus I) led to no systematic changes in vocal compensations for pitch perturbations. Likewise, the cortical N1 and P2 responses to pitch perturbations in voice auditory feedback did not vary as a function of cerebellar TBS modality. These findings provide the first neurobehavioral evidence that the left cerebellum is not causally involved in auditory feedback control of vocal production, suggesting that this region may not be a significant contributor to auditory-vocal integration.

Multiple lines of evidence have demonstrated cerebellar involvement in a variety of language functions (Justus, 2004; Durisko and Fiez, 2010; Lesage et al., 2012), and the majority of these studies have shown a right-lateralized linguistic cerebellum (Marien et al., 2014). A growing body of literature has also shown significant contributions of the right cerebellum to speech production (Riecker et al., 2000; Riva and Giorgi, 2000; Tourville et al., 2008; Stoodley, 2012; Ziegler and Ackermann, 2017). In particular, increased activity in the right cerebellum was found during the production of compensatory speech responses to *F*_1_ perturbations (Tourville et al., 2008). More recently, a number of tDCS and cTBS studies provide causal evidence that links the right cerebellum to auditory feedback control of vocal production (Lametti et al., 2018; Peng et al., 2021; Lin et al., 2022). For example, following cTBS over the right cerebellar lobule VII (Crus I), patients with SCA exhibited smaller vocal compensations for pitch perturbations paralleled by larger P1 and P2 responses and smaller N1 responses when compared to sham stimulation (Lin et al., 2022). In contrast, only a few studies reported activation of the left cerebellum during speech production (Chen and Desmond, 2005; Bohland and Guenther, 2006) and simple singing (Perry et al., 1999). As well, increased activation of bilateral cerebellum was found when perturbations of jaw movement prompted the generation of compensatory speech motor commands (Golfinopoulos et al., 2011). In the present study, however, we did not find systematic changes in the vocal or N1/P2 responses to pitch perturbations following cTBS or iTBS over the left cerebellar lobule VII (Crus I), indicating the lack of a causal link between the left cerebellum and auditory-vocal integration. Along with previous findings showing a causal role of the right cerebellum for vocal pitch regulation (Peng et al., 2021; Lin et al., 2022), our findings lend support to a hypothesis that the cerebellum may contribute to the functioning of the neural mechanisms that support auditory feedback control of vocal production in a hemispheric-specific manner. In line with this hypothesis, other studies found that cTBS over the right cerebellum led to impaired verbal working memory and reduced accuracy in lexical tasks whereas cTBS over the contralateral region did not (Argyropoulos, 2011; Tomlinson et al., 2014).

Notably, the cerebellum is anatomically subdivided into a discrete set of regions (lobules I-X) that are associated with a diverse set of motor and cognitive tasks (King et al., 2019), suggesting a lobule-specific relationship between cerebellar tissues and behavioral performance. During speech/language processing, for example, it has been suggested that the superior cerebellum (lobule VI and Crus I) is involved in articulatory control while the inferior posterior cerebellum (lobules VIIb and VIII) is involved in verbal working memory (Chen and Desmond, 2005; Frings et al., 2006). In the context of speech motor control, activity in the right-lateralized cerebellar lobule VIIIA was identified when speech *F*_1_ was perturbed in auditory feedback (Tourville et al., 2008), while somatosensory perturbations to jaw movements activated the bilateral cerebellar lobule VIII during speech production (Golfinopoulos et al., 2011). The absence of modulatory effects of cTBS or iTBS over the left cerebellar lobule VII (Crus I) on vocal pitch regulation observed in the present study, therefore, cannot rule out the possibility that the left cerebellum may be involved in auditory-vocal integration in a lobule-specific manner. Stimulating other cerebellar regions such as the lobule VI or VIII, which has been implicated in speech production (Perry et al., 1999; Golfinopoulos et al., 2011), is warranted to elucidate the potential role of the left cerebellum in vocal feedback control in the future studies.

On the other hand, different types of TMS coils such as the figure-of-eight, double cone, and batwing coils have been chosen to probe cerebellar functions according to the depth of cerebellar tissues (Hardwick et al., 2014; Vinas-Guasch et al., 2022). Hardwick et al. (2014) compared the effectiveness of cerebellar stimulation across the three coil designs and found that the double-cone and batwing coils, but not the figure-of-eight coil, effectively stimulated the cerebellar lobules V and VIII for eliciting cerebellar-brain inhibition. In light of this finding, it is suggested to stimulate the superficial cerebellar tissues using the figure-of-eight coil and the deeper-lying targets using the double cone or batwing coil (Hardwick et al., 2014). However, there is evidence for the use of the figure-of-eight coil to effectively stimulate the lobule VI (Tomlinson et al., 2014) or VIII (Popa et al., 2010). Therefore, further investigations should be careful to choose coil designs suitable for stimulating cerebellar tissues with different depths. Note that increased depth of cerebellar stimulation with the double cone or batwing coil is achieved at the expense of focality (Deng et al., 2013), which is in contrast with the precise stimulation of cerebellar regions using the figure-of-eight coil guided by the neuronavigation system (Hurtado-Puerto et al., 2020).

Two limitations in the present study should be acknowledged. First, the present study used sham stimulation as a control condition, which potentially allows participants to distinguish between sham and active stimulation due to non-specific sensory effects of TMS (e.g., click sounds, skin sensation) (Duecker and Sack, 2015). Future studies are thus needed to compare the sham approach with other control conditions such as stimulating the vertex (Cattaneo et al., 2014; Jung et al., 2016) or a site that is unrelated to the task (Gatti et al., 2020; Ramos Nunez et al., 2020) to determine the optimal control strategy. On the other hand, the data from participants following cTBS, iTBS, and sham stimulation over the contralateral right cerebellum were not obtained, which would be helpful to determine whether there is a cerebellar lateralization in vocal motor control. Despite these limitations, the present study presents the first evidence that the left cerebellar lobule VII (Crus I) does not exert a causal influence on vocal pitch regulation, offering a starting point to investigate the role of the left cerebellum in vocal motor control.

## Data availability statement

The raw data supporting the conclusions of this article will be made available by the authors, without undue reservation.

## Ethics statement

The studies involving human participants were reviewed and approved by Institutional Review Board of The First Affiliated Hospital of Sun Yat-sen Unsiversity. The patients/participants provided their written informed consent to participate in this study.

## Author contributions

DL, MC, PL, and HL contributed to the design of the study. DL, MC, QL, TL, XC, XW, and JL contributed to the acquisition and analysis of data. DL, MC, PL, and HL contributed to drafting the manuscript and preparing the figures. All authors have reviewed and approved the contents of the manuscript.
